# Somatic mosaicism in schizophrenia brains reveals prenatal mutational processes

**DOI:** 10.1126/science.adq1456

**Published:** 2024-10-10

**Authors:** Eduardo A. Maury, Attila Jones, Vladimir Seplyarskiy, Thanh Thanh L. Nguyen, Chaggai Rosenbluh, Taejong Bae, Yifan Wang, Alexej Abyzov, Sattar Khoshkhoo, Yasmine Chahine, Sijing Zhao, Sanan Venkatesh, Elise Root, Georgios Voloudakis, Panagiotis Roussos, Brain Somatic Mosaicism Network, Peter J. Park, Schahram Akbarian, Kristen Brennand, Steven Reilly, Eunjung A. Lee, Shamil R. Sunyaev, Christopher A. Walsh, Andrew Chess

**Affiliations:** 1 Division of Genetics and Genomics, Manton Center for Orphan Disease, Boston Children’s Hospital, Boston, MA 02115, USA; 2 Bioinformatics & Integrative Genomics Program and Harvard/MIT MD-PHD Program, Harvard Medical School, Boston, MA 02115, USA.; 3 Program in Medical and Population Genetics, Broad Institute of MIT and Harvard, Cambridge, MA, USA; 4 Department of Cell, Developmental & Regenerative Biology, Icahn School of Medicine at Mount Sinai, New York, NY 10029, USA.; 5 Department of Genetics and Genomic Sciences, Icahn School of Medicine at Mount Sinai, New York, NY 10029, USA; 6 Department of Biomedical Informatics, Harvard Medical School, Boston, MA 02115, USA; 7 Division of Genetics, Brigham and Women’s Hospital, Harvard Medical School, Boston, MA 02115, USA; 8 Department of Genetics, Yale School of Medicine, New Haven, CT 06520, USA; 9 Department of Psychiatry, Yale School of Medicine, New Haven, CT 06520, USA; 10 Department of Quantitative Health Sciences, Center for Individualized Medicine, Mayo Clinic, Rochester, MN 55905, USA; 11 Department of Neurology, Brigham and Women’s Hospital, Boston, MA 02115, USA; 12 Center for Disease Neurogenomics, Department of Psychiatry, Icahn School of Medicine at Mount Sinai, New York, NY 10029, USA; 13 A list of authors and their affiliations appears in Table S1; 14 Department of Psychiatry and Neuroscience, Friedman Brain Institute, Mount Sinai, New York, NY 10029, USA.; 15 Department of Neuroscience, Friedman Brain Institute, Mount Sinai, New York, NY 10029, USA.; 16 Departments of Pediatrics and Neurology, Harvard Medical School, Boston, MA 02115, USA; 17 Howard Hughes Medical Institute, Boston Children’s Hospital, Boston, MA 02115, USA

## Abstract

Germline mutations modulate the risk of developing Schizophrenia (SCZ). Much less is known about the role of mosaic somatic mutations in the context of SCZ. Deep (239x) whole-genome sequencing (WGS) of brain neurons from 61 SCZ and 25 controls postmortem identified mutations occurring during prenatal neurogenesis. SCZ cases showed increased somatic variants in open chromatin (p <0.0001), with increased mosaic CpG transversions (CpG>GpG) and T>G mutations at transcription factor binding sites (TFBS) overlapping open-chromatin, not seen in controls. Some of these variants alter gene expression, including SCZ risk genes and genes involved in neurodevelopment. Although these mutational processes can reflect difference in factors indirectly involved in disease, increased somatic mutations at developmental TFBS could also potentially contribute to SCZ.

Schizophrenia (SCZ) has a substantial genetic component, with common variants (minor allele frequency >1%) of individually small effect, as well as rare copy number variants (CNV) and single nucleotide variants (SNV) with larger effects, all contributing to genetic risk (1). Somatic variants, which occur throughout development and hence are present in a fraction of cells in the body (2, 3), are familiar drivers of cancer, but are increasingly recognized as contributing to neurodevelopmental conditions including focal epilepsy (4, 5) and autism spectrum disorders (ASD) (6, 7). Recent work implicates somatic CNV in a fraction of SCZ cases (8), whereas the contribution of somatic SNV (sSNV) remain largely unexplored.

## Study design and variant discovery

**Fig. 1.**
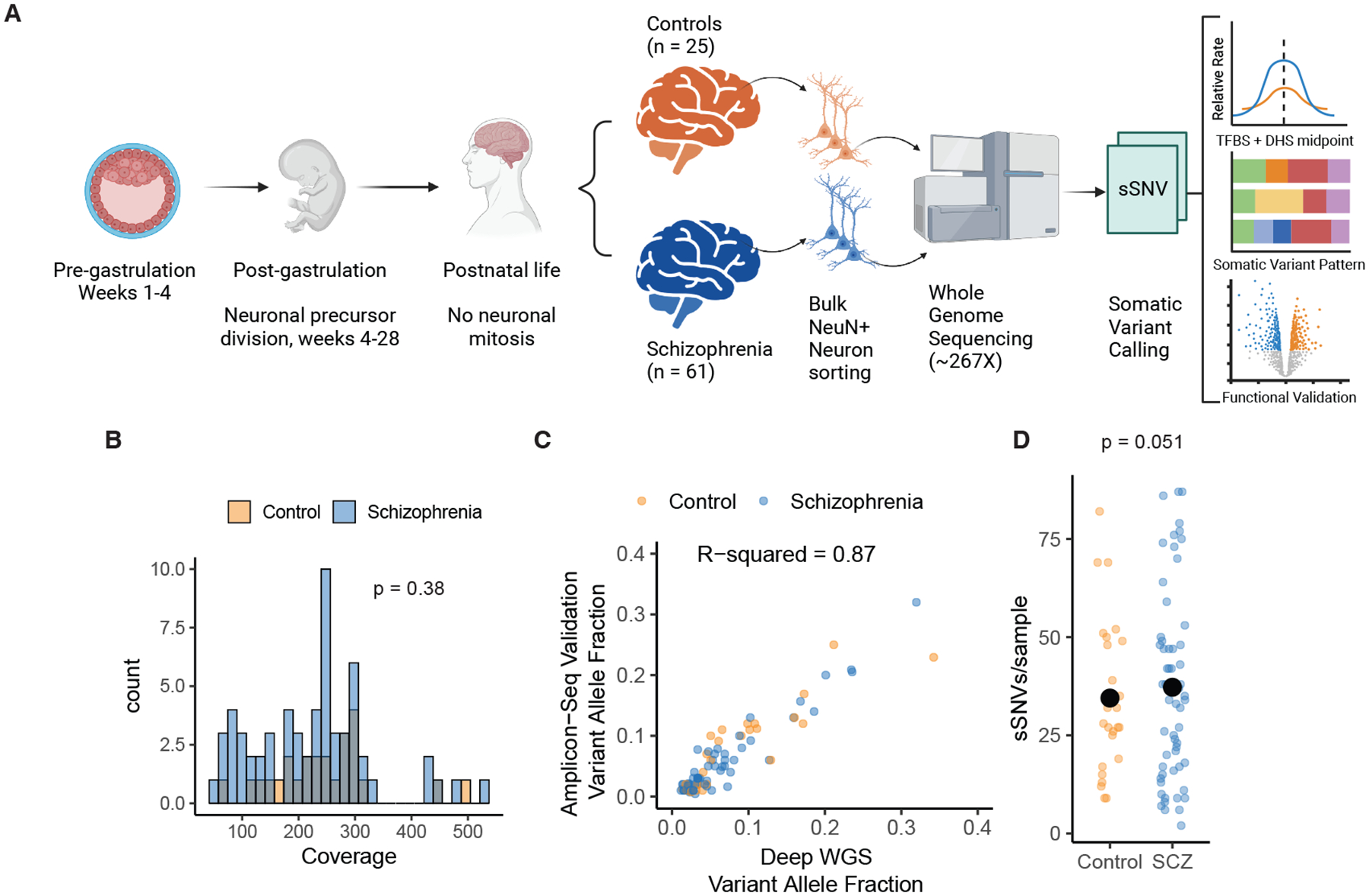
Experimental design and orthogonal validation. A) Schematic of experimental and analysis design. Notably, neuronal clonal somatic mutations that are shared across neurons originate during prenatal brain development; occurring either before organogenesis (pre-gastrulation) or during neuronal proliferation during neurogenesis, resulting in somatic variants present in cells across multiple tissues. Mutations occurring postnatally in neurons are not clonal and hence undetectable with this method. B) Histogram of average sequencing coverage for schizophrenia cases and control samples. C) Scatter plot of Deep WGS variant allele fraction (VAF) for variant submitted for validation and the VAF from the validation amplicon sequencing from SCZ and controls samples. R-squared value was computed from ordinary linear regression model. D) Scatter plot of number of sSNV per sample for schizophrenia cases and control *after* removal of an outlier SCZ case with 188 sSNVs. Large black points represent the sample medians. The p-value was calculated using permutation based negative binomial step-regression (see Methods).

We analyzed somatic variants directly from postmortem brain, using deep WGS of DNA extracted from NeuN+ neurons of dorsal lateral prefrontal cortex (DLPFC) from 61 individuals with a diagnosis of SCZ and 25 neurotypical controls (Fig. 1A, Table S2, Methods) to specifically capture mutations occurring during early prenatal development. Since neocortical neurons are all post-mitotic by ~30 gestational weeks (9), somatic mutations clonally shared by neurons occur in progenitor cells prior to 30 weeks, and are not confounded by post-gestational clonal mutations.

Subjects were of European and African ancestry based on principal component analysis (Fig. S1A). Polygenic risk score (PRS) normalized by ancestry revealed that, as expected, individuals with SCZ had a higher PRS for disease than controls (Kolmogorov-Smirnov test p = 0.0086, Fig. S1B). Brains tissue was homogenized and nuclei stained for NeuN, and subjected to FANS using standard methods (10). DNA extracted from 500,000–1,000,000 nuclei was sequenced without amplification (Methods). Median genome coverage of ~239X showed no significant difference in coverage between cases and controls (Wilcox Ranksum Test p = 0.38, Fig. 1B).

Somatic SNVs were identified using best practices of the Brain Somatic Mosaicism Network (BSMN), which offers high sensitivity (11, 12). The final call-set of 3,286 sSNV (2,424 in SCZ and 862 in controls, Table S3) showed variant allele fractions (VAF) from 0.92% to 39.7%. We randomly selected 111 variants to validate with 96 having enough coverage for orthogonal amplicon-based sequencing (Methods). 90/96 sSNV validated (94%) with VAFs highly correlated with WGS estimates (R-squared = 0.87, Fig. 1C), and no differences in validation between cases (62 validated, 5 not) and controls (28 validated, 1 not) (Fisher exact test p=0.66). One outlier SCZ sample showed 188 mutations without technical anomalies or unconventional nucleotide substitution patterns (13); since this high mutational burden could dominate downstream statistics, the sample was excluded from all counts and further analyses.

## Genome-wide sSNV burden in cases and controls

After exclusion of the outlier SCZ sample, genome-wide sSNV counts in remaining SCZ cases averaged 37.3 per sample compared to 34.5 in controls, which did not achieve statistical significance using permutation-based negative binomial regression (p = 0.051, Fig. 1D, Methods). For each permutation we randomly shuffled diagnosis labels and ran a forward negative binomial step-regression model to account for ancestry principal components, sex assigned at birth, and technical covariates (sequencing facility, coverage, year of autopsy, age of death, cause of death, postmortem-interval, and institution where diagnosed). Regression analysis was performed on 45 SCZ cases and 19 controls with information across all covariates, including ancestry principal components (Table S4). As expected, age was not associated with higher sSNV/sample (p>0.05, Fig. S1C), emphasizing that identified clonal variants occurred prenatally in neuronal precursors, remaining static after birth. Although permutation provides uninflated p-values, power analysis suggests that with mutation rates increased < 1.7-fold in SCZ versus controls, as observed here, this test provides low power to detect significant differences (Fig. S1D, E).

One SCZ case showed a somatic copy number variant (sCNV) overlapping intron 1 and potentially exon 2 of *SORCS2* (Fig. S2A, B), implicated in attention-deficit hyperactive disorder (ADHD), and bipolar disorder (14, 15), though roles of *SORCS2* in SCZ are not established. sSNV were not enriched in GWAS loci associated with SCZ (binomial regression, p = 0.936). Exonic sSNV (87 total, 2.6%, including the outlier sample) were equally common in cases (1.02 per individual) and controls (1.00 per individual, p =1, Fisher Exact test), and we did not detect somatic stop-gain, splice-site altering, or missense variants at genes implicated in SCZ in germline *de novo* or rare variant studies (16) in our small sample (Table S3). However, we did find a stop-gain T>G sSNV on exon 1 of *STX12/13* (chr1:28099835, T>G, p.L6*), a highly constrained gene (probability of heterozygous loss intolerance, pLI, of 0.96 (17)) that encodes an endosomal synaptic transport protein.

## Higher sSNV rate at active TFBS in SCZ

**Fig. 2.**
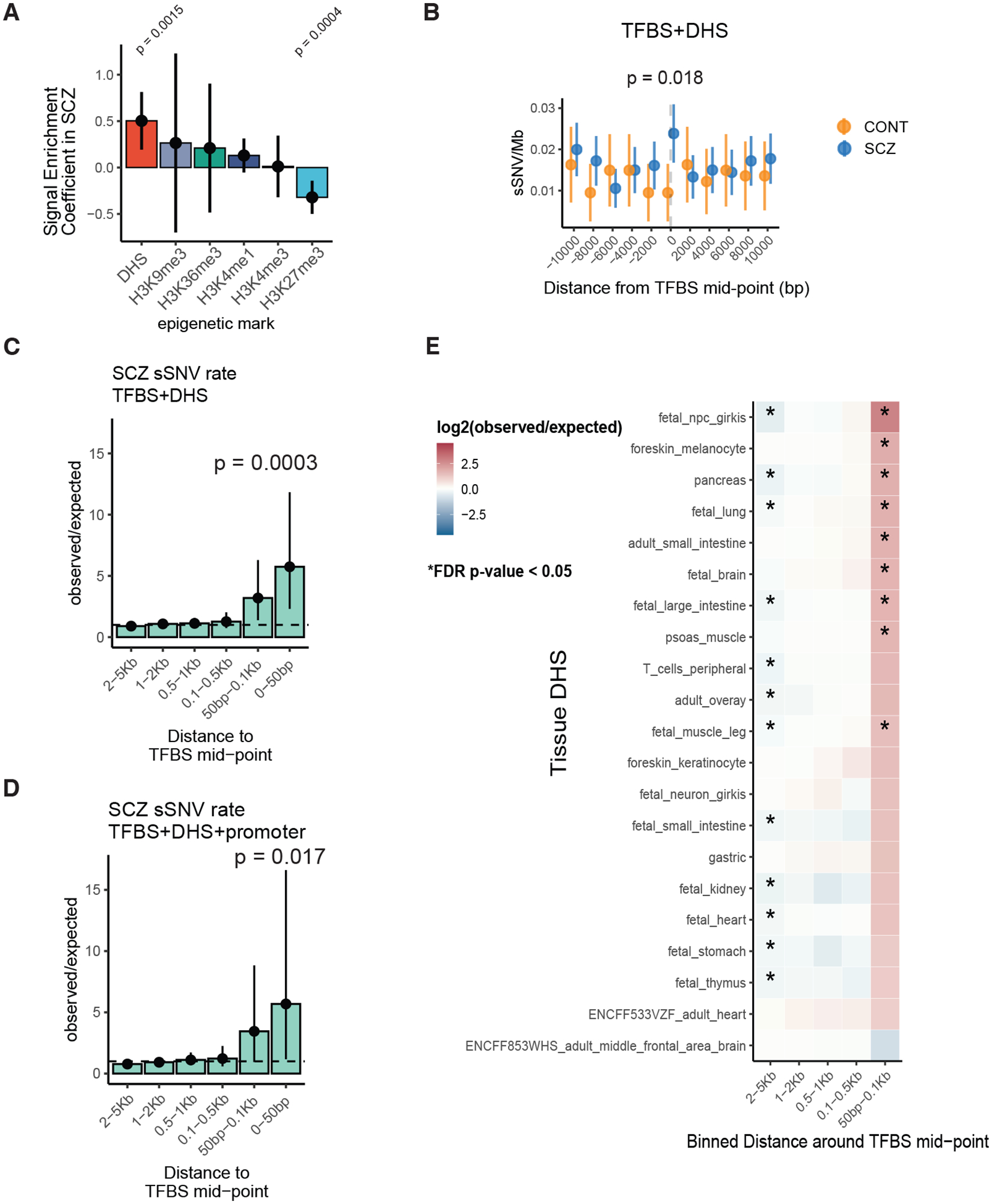
Increased sSNV rate at developmentally active transcription factor binding sites (TFBS in SCZ. A) Bar plot of binomial regression interaction term between epigenomic tracks and disease status. Positive values indicate enrichment in SCZ and negative values indicate depletion. Line ranges indicate 95% confidence intervals from binomial regression. B) Somatic SNV rate at +/− 10Kb region from active TFBS in fetal brain (TFBS+DHS) in SCZ and controls. C, D) Bar plot of observed over expected mutation rate at binned regions around TFBS in SCZ. E) Heatmap of rate ratios in SCZ at TFBS using different DHS tracks. For B, C, D, and E p-values and confidence intervals were calculated using Poisson tests. For E, stars indicate statistical significance at the FDR adjusted p < 0.05 level.

Analysis of sSNV distribution across the genome, using fetal brain tracks from Roadmap Epigenomes (18), revealed increased sSNV in open chromatin regions in SCZ compared to controls. Previous comparison of ASD to controls showed enrichment of sSNV at open chromatin regions (7, 12). We found higher sSNV rates in SCZ versus controls at fetal brain DNase hypersensitivity sites (DHS), indicative of open chromatin (binomial regression, p = 0.0015, Fig. 2A). Conversely, we found lower sSNV rate in SCZ at H3K27me3 regions, associated with downregulation of genes and closed chromatin (19) (binomial regression, p = 0.0004, Fig. 2A). To ensure that the genome-wide overdispersion of sSNV did not inflate these statistics, we obtained a null p-value distribution by permuting diagnosis labels. This empiric null p-value distribution was very close to the expected null, suggesting robustness to overdispersion (Fig. S3A). We did not detect case-control differences in sSNV rate at regions of increased fetal brain gene expression, nor a systemic transcriptional strand bias (Fig. S3B). We also did not find significant association between sSNV rate and replication timing or replication fork direction (Fig. S3C).

Previous studies in cancers observed enriched sSNV at active TFBS overlapping DHS, due to hindrance of DNA repair by bound transcription factors (TFs) (20–22). To test whether a similar phenomenon could explain the local increase in sSNV at DHS regions in SCZ, we calculated sSNV rates near the midpoint of TFBS, accounting for the number of genomes and sites sampled in each SCZ case (Methods). We aggregated the hg19 TFBS BED files from Vorontsov et al (23) using human TF tracks with highest reliability and reproducibility (A tracks). These tracks aggregate across experimental designs and tissues, so that they are not tissue-specific. We used the top 10% of DHS intensity from fetal brain tracks of Roadmap Epigenomes (18) to obtain likely active TFBS. We observed increased sSNV near (+/− 1Kb) the midpoint of active TFBS in SCZ compared to controls (Poisson test, RR = 2.51 [1.12:6.62], p = 0.018, Fig. 2B). Results were robust to DHS intensity threshold (Fig. S4A, B). No individual TF achieved statistical significance after multiple hypothesis correction.

Further genome-wide analysis of SCZ sSNV, comparing rates near active TFBS to expected genome-wide rates after accounting for trinucleotide context, revealed 5.74-fold enrichment within 50bp from the TFBS mid-point (p = 0.0003, Fig. 2C), and 5.68-fold enrichment near promoters (p = 0.017, Fig. 2D), with effects fading >100bp from the TFBS midpoint, suggesting highly localized mutational processes. Similar enrichment was observed across DHS intensity cut-offs, with increasing effect sizes with higher DHS signal (Fig. S4C, D). This rate comparison is across genomes of SCZ only, excluding effects of sequencing or hereditary differences. We observed enrichment of sSNV at TFBS across DHSs from multiple tissues, developmental stages and embryonic germ layers (including 10 fetal and 10 adult, Table S5, Fig. 2E), suggesting a pattern that is developmental, but not tissue-specific. No similar enrichment was observed in controls.

## Specific sSNV patterns at TFBS in SCZ

**Fig. 3.**
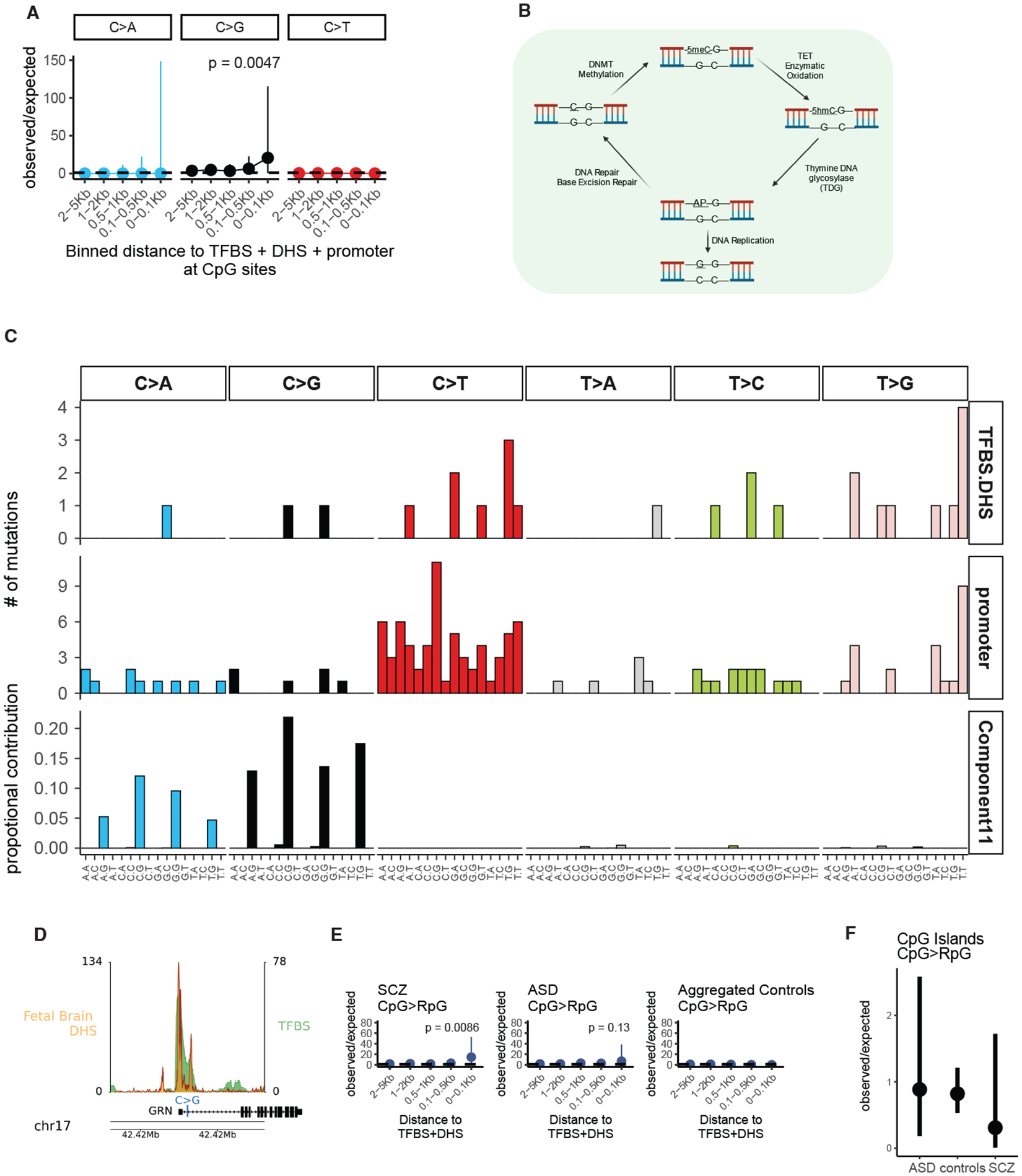
Increased somatic CpG transversions at active transcription factor binding sites in SCZ. A) Forest plots of rate ratios in SCZ of different base changes in active TFBS at CpG sites. B) Trinucleotide context plot of sSNV in schizophrenia at active TFBS and promoter sites, and CpG transversion signature Component 11(24). C) Schematic of enzymatic demethylation mechanism resulting in CpG transversions. Abbreviations: 5meC, 5-methyl-cytosine; 5hmc, 5-hydroxymethyl-cytosine; AP abasic site. D) Illustration of promoter CpG>GpG mutation of *GRN* gene with DHS and TFBS tracks. E) Forest plots of observed vs expected CpG transversions at active TFBS in promoter regions from schizophrenia, autism spectrum disorder, and aggregated control. F) Forest plot of the relative observed vs expected CpG transversions at CpG islands across diagnostic categories. For panels A, E, and F, p-values and 95% confidence intervals were computed using a Poisson test.

Two specific base substitution patterns were observed in SCZ but not in controls. Somatic SNVs at CpG sites showed 24.0-fold enrichment of CpG>GpG substitutions at active TFBS at promoters compared to the expected C>G genome-wide rate accounting for trinucleotide context (2 observed, 0.083 expected)(95% CI [2.90:86.5], p=0.0047, Fig. 3A, Fig. S5A). C>G and C>A transversions at CpG contexts characterize a known mutational process (Component 11, Fig. 3B) (24) reflecting enzymatic demethylation, which involves resection of oxidated methyl-cytosine, creating an abasic site (25) (Fig. 3C). Replication of the abasic site before repair creates CpG transversions (26). One CpG>GpG variant in SCZ was near the promoter of *GRN,* encoding the essential, dosage-sensitive protein, progranulin (Fig. 3D). *GRN* haploinsufficiency causes frontotemporal dementia in adults and pediatric neuronal degeneration (27) and has been reported in SCZ (28).

In addition to enrichment of CpG transversions at active TFBS in SCZ (Observed/Expected 14.5, 95% CI [1.76:52.57], p=0.0086, Fig. 3E), we observed a similar trend in bulk brain DNA from ASD cases analyzed previously (7)(Observed/Expected 6.92, 95% CI [0.18:38.6], p=0.13, Fig. 3E). In contrast, we did not find CpG transversions ≤ 100bp from the mid-point of TFBS in 1) our control samples, 2) control samples from the WGS ASD cohort (7), nor 3) a recent non-diseased twin study (Fig. 3E)(29), so that CpG transversions at TFBS were increased in SCZ versus aggregated controls (p=0.013, binomial test). In contrast, somatic CpG transversions at CpG islands showed similar rates in SCZ, ASD, and controls (Fig. 3F), suggesting that CpG transversions in CpG islands are not disease-associated.

**Fig. 4.**
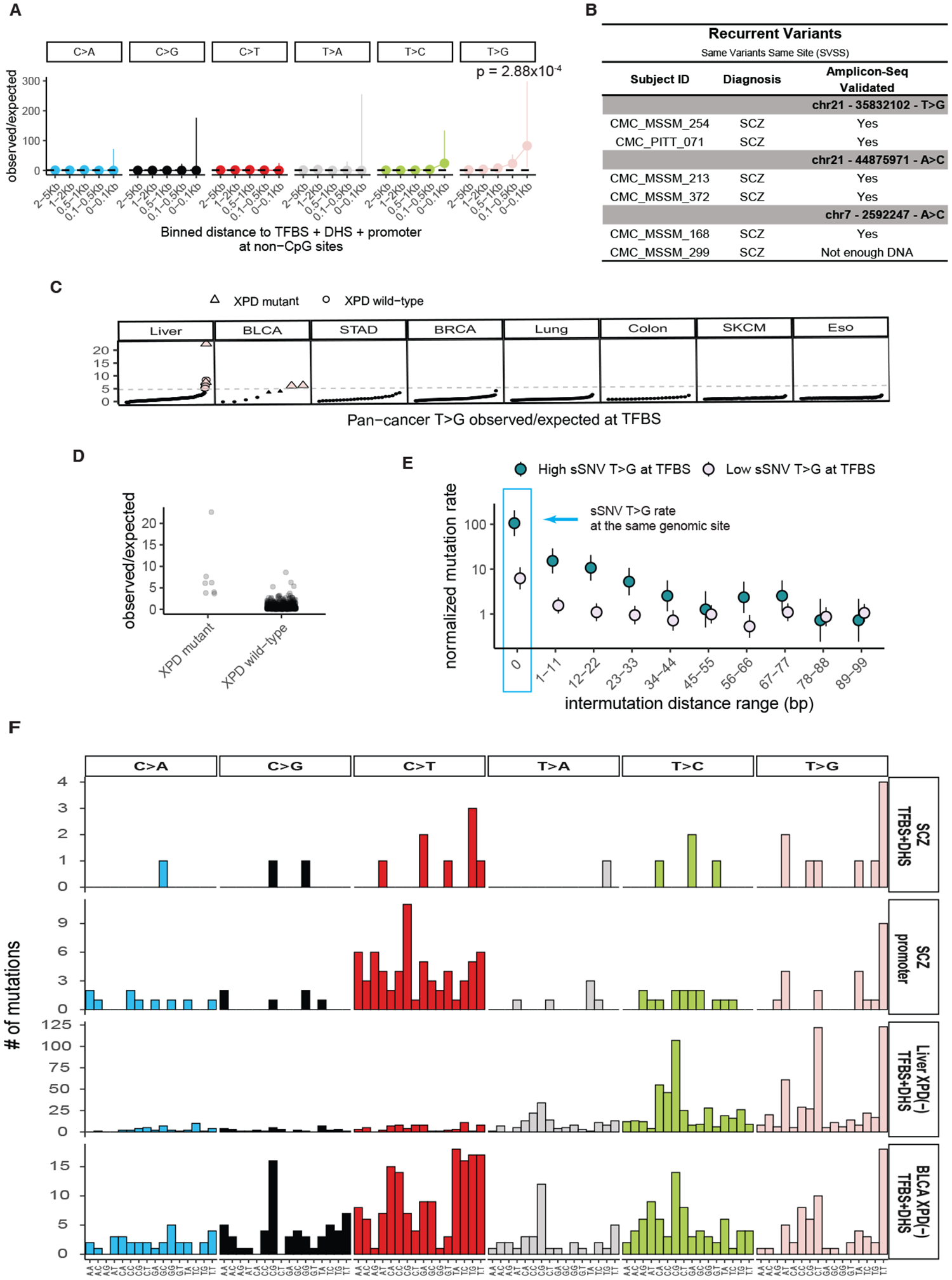
Increased somatic T>G substitutions at active TFBS in SCZ and cancer samples. A) Forest plots of rate ratios in SCZ of different base changes in active TFBS at promoter regions at non-CpG sites. P-values and 95% confidence intervals were computed using a Poisson test. B) List of T>G variants occurring at the same genomic position. C) T>G sSNV observed vs. expected mutation rate at TFBS across various cancer types. Samples on the x-axis are sorted based on observed/expected ratios for each cancer category. Pink data points indicate samples with enriched T>G burden at TFBS. Triangles indicate samples with *XPD* mutation. D) Observed over expected ratio of T>G sSNV at TFBS in cancer samples carrying *XPD* mutations, vs non-carriers. E) Forest plot of sSNV rate in Liver and Bladder cancers stratified by enrichment of T>G mutations at TFBS (pink data points from panel C). F) 96 trinucleotide context of SCZ sSNV at active TFBS (TFBS+DHS) and at promoter regions, along with liver and bladder cancer sSNV from samples with *XPD* dysfunction at active TFBS. The corresponding tissue DHS track for each cancer type was obtained from the ENCODE database (Table S5).

Relative rates of sSNV across base changes at non-CpG sites in SCZ samples, although accounting for trinucleotide context, showed highly localized increase in T>G substitutions within 100 bps from the TFBS midpoint versus genome-wide expectation (observed= 3, expected = 0.09, observed/expected =34.3 95% CI [7.08:100.3], p = 1.04×10^−4^, Fig. S5B), which was further enhanced near promoters (observed = 2, expected = 0.024, observed/expected = 82.6, 95% CI [10.0:298.4], p = 2.88×10^−4^, Fig. 4A). T>G sSNVs in active TFBS showed significant enrichment in cases versus the aggregated-control sample above (p = 0.0032, binomial test). Genome-wide T>G mutations also represented a higher proportion of sSNV in SCZ versus control (Permutation Fisher Exact Test, OR= 2.23, p <0.0001, Fig. S6).

Of note, 3 unrelated pairs of SCZ cases showed the exact same T>G substitution at the exact same genomic position (Fig. 4B), which we call same variant same site (SVSS) recurrence. We saw no somatic T>G SVSS recurrence in controls or other deep WGS samples including ASD(7). We confidently validated 2/3 pairs of T>G variants through orthogonal amplicon sequencing; for the third pair we only had DNA from one individual, which showed positive validation (Fig. 4B). The exceedingly low probability (Poisson Test, p = 1.22×10^−11^) of observing 3 recurrent sSNVs by chance suggests that mutational processes driving these T>G mutations are highly localized, with T>G mutational hotspots showing an estimated sSNV rate ~1.44×10^5^ times the expected genome wide rate (Methods).

Analysis of T>G mutations across the Pan Cancer Analysis of Whole Genomes (PCAWG (30)) suggested potential mechanisms for T>G mutagenesis at TFBS. We found similarly high rates of T>G mutations at TFBS in a subset of liver and bladder cancer samples, with 6 liver and 2 bladder cancer samples showing strong T>G mutation enrichment at TFBS (>5-fold, red circles Fig. 4C). Similar to SCZ, these liver and bladder cancer samples showed SVSS recurrence, which was enriched in samples with high sSNV rate at TFBS versus those with low sSNV rate at TFBS (~100- vs ~10-fold respectively, Fig. 4D, E). Three of the six liver and both bladder samples showing SVSS recurrence carried somatic missense mutations in *XPD*, a key DNA repair gene, in line with observations that *XPD* dysfunction can increase sSNV at TFBS (31). Cancer samples with *XPD* mutations were also enriched in T>G mutations at TFBS versus non-carriers (Wilcoxon rank-sum test p-value = 6.3×10^−6^, Fig. 4D).

The trinucleotide mutational spectra at TFBS of liver and bladder cancers showing high TFBS mutation rates and *XPD* deficiency were very similar to that of SCZ at active TFBS (Fig. 4F). Despite remarkably converging patterns between liver/bladder cancer and SCZ, we did not find *XPD* somatic or germline mutations in SCZ, nor T>G mutations in common, though *XPF* (ERCC4), encoding another core nucleotide excision repair gene, is a reproducible SCZ GWAS hit (32, 33). Thus, mosaic SCZ mutations may be driven by factors mimicking XPD dysfunction, such as other factors inhibiting DNA repair; though perhaps the similar mutation spectra are coincidental.

## Functional interrogation of somatic variants

**Fig. 5.**
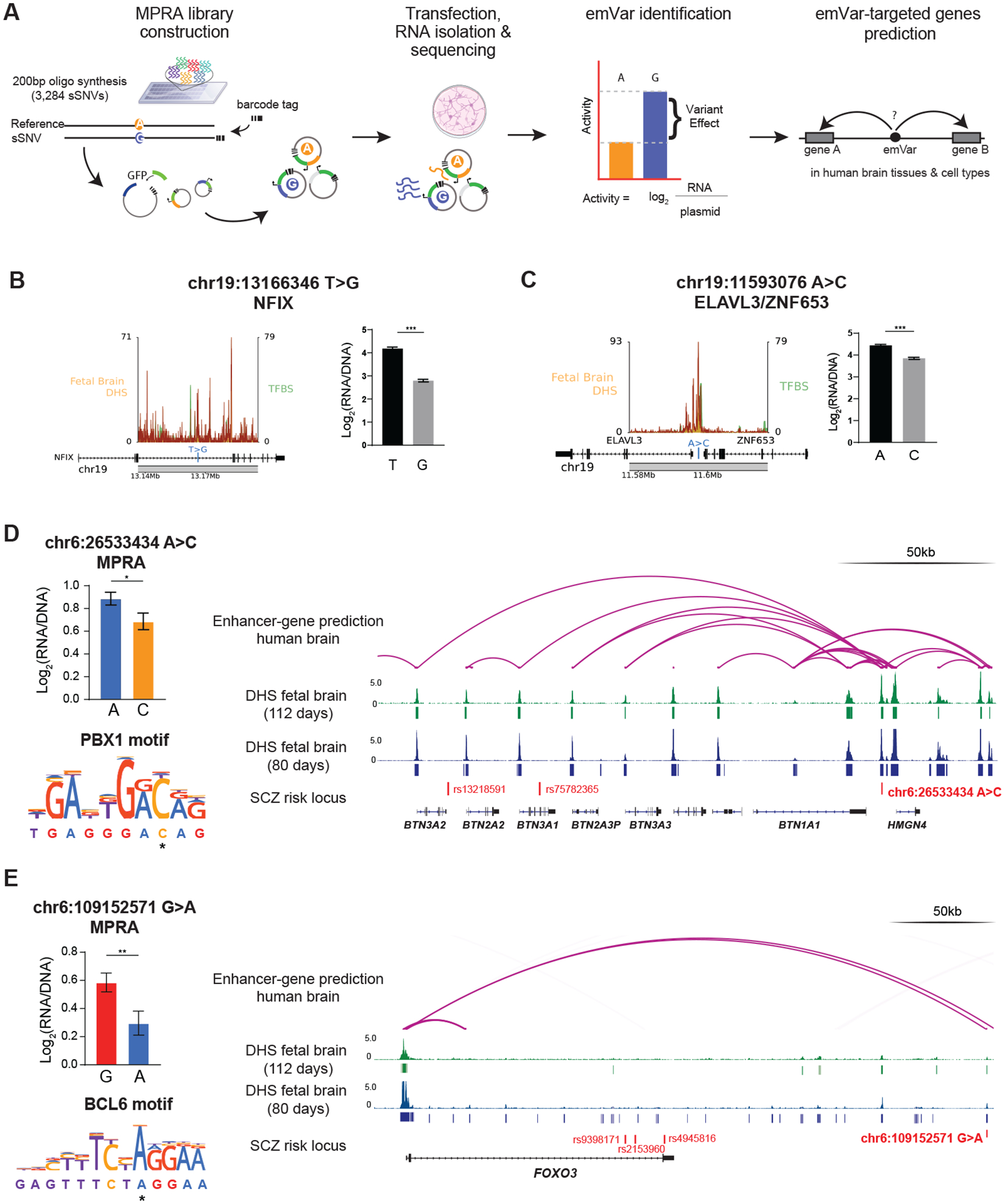
Transcriptional impact of early developmental somatic variants in SCZ and control individuals. A) Schematic of MPRA experimental design. B, C) Schematic of T>G sSNV occurring near developmental genes *NFIX* and *ELAVL3*/*ZNF63,* with DHS and TFBS tracks. MPRA bar plots represent expression levels from each allele in MPRA. P-values represent Benjamini-Hochberg-corrected Wald’s test between the log ratios of the reference and alternative alleles. D) & E) MPRA results, motif break prediction, and integrative genomic viewer of enhancer-gene linkage map for somatic-emVars targeting known SCZ risk genes. MPRA bar plots represent expression levels from each allele in MPRA, and P-values represent Benjamini-Hochberg-corrected Wald’s test between the log ratios of the reference and alternative alleles. DHS tracks for human fetal brain tissues at different stages are from the ENCODE portal.

We used massively parallel reporter assays (MPRA) in a human neuroblastoma cell line (SK-N-SH, Methods) to assess gene regulatory impacts of the full set of sSNV identified in cases and controls (Fig. 5A, Table S3, S6). MPRA regulatory activity measurements were highly reproducible (five replicates’ pairwise Pearson’s r ≥ 0.99, Fig. S7) and recapitulated known SK-N-SH positive and negative controls (Fig. S8)(34, 35). The rate of somatic mutations causing significant expression modulation (emVars) did not differ by diagnosis (Permutation Fisher Exact test p=0.49). Variants were equally likely to up- or down-regulate expression (Fig. S9A). T>G transitions were nominally more likely to be emVars in SCZ versus controls (Permuted Fisher exact test p = 0.03, OR=2.6, Fig. S9B, C, D, E) but no mutation type was enriched after multiple hypothesis correction. Some emVars were located at TFBS within DHS near neurodevelopmental genes. For example, emVar chr19:13166346 T>G decreases regulatory activity (BH-correct Wald’s test p<0.0001, Log2FC = −1.36) and is near NFIX (Fig. 5B, S9D), in which heterozygous loss of function mutations cause Malan syndrome, characterized by brain overgrowth and behavioral abnormalities (36). Another emVar, chr19:11593076 A>C (BH-corrected Wald’s test p<0.0001, Log2FC= −0.57) is near ELAVL3, a neuron-specific RNA-binding protein that regulates glutamate neurotransmission and neuronal excitability (37, 38) (Fig. 5C, S9D).

We predicted brain-specific genes targeted by regulatory elements harboring somatic emVars using gene-enhancer linkage maps (Table S9, Method) (39, 40), linking 88 emVars to 247 candidate target genes, with some sSNVs linking to multiple genes (range: 2–13 targets). Two somatic emVars target seven genes overlapping SCZ risk loci (Fig. 5D, E, Methods). These variants had the same direction of effect in all tested contexts in MPRA and caused regulatory disruption across most windows (Fig. S9D). In particular, emVar chr6:26533434 A>C, a T>G that downregulates activity (BH-corrected Wald’s test p < 0.05, Log2FC = −0.18), creates a predicted binding site for PBX1 (p < 0.0001, allele difference = 0.99), a repressive regulator of neuron development (41)(Fig. 5D). The variant maps its gene-enhancer activity to the genes BTN1A1, BTN2A3P, BTN3A1, BTN3A2, BTN3A3, and HMGN4 within the major histocompatibility complex class I region (Fig. 5D), a locus reproducibly associated with SCZ (42, 43). The emVar chr6:109152571 G>A also decreases transcription (BH-corrected Wald’s test p<0.001, Log2FC= −0.27) and is predicted to loop to the *FOXO3* promoter (Fig. 5E, Fig. S9D), associated with schizophrenia. The variant creates a predicted binding site for BCL6 (p<0.0001, allele difference = 1.52), a direct repressor of *FOXO3* (44). Together, *BCL6* and *FOXO3* reciprocally regulate neural stem cell proliferation and differentiation(45).

## Discussion

Although our data are limited by sample size, they suggest mutational models that could explain distinctive sSNV patterns in SCZ. CpG transversions make up ~2.4% of all mosaic mutations in brain tissue, potentially originating in the early zygote shortly after fertilization, when global DNA demethylation of the paternal and maternal genomes restores totipotency at the maternal-to-zygotic transition (24, 25, 46). Alterations in this process, either endogenous or exogenous, would predispose to somatic CpG transversions. The high VAF of CpG transversions at TFBS (average VAF = 13%) is consistent with this very early occurrence. We speculate that the last step of demethylation could be obstructed by TF binding, analogous to interference between TF binding and DNA repair in cancer (20–22), where enrichment of sSNV at active TFBS has been attributed to steric interference of TFs with the repair apparatus. Comparison of mosaic mutations between SCZ and controls is remarkable because the overall burden in CpG transversions is higher than in germline for both cases and controls (24), but effects of TF binding are unique to neuropsychiatric disease.

Somatic T>G mutations may reflect a *XPD* dysfunction-like mechanism as suggested by the similarity in mutational patterns at TFBS between SCZ and cancers deficient in *XPD*. This mechanism could produce preferential sSNV accumulation at active TFBS due to hindrance of DNA repair by TFs bound to damaged DNA (21). On the other hand, although we did not find deleterious somatic or germline mutations in *XPD* in any SCZ samples, we cannot exclude altered nucleotide excision repair or *XPD* expression by other mechanisms. The root cause of T>G mutations, even in cancer, is unclear. They have been proposed to reflect oxidative damage to deoxyribonucleotides in rapidly dividing cells (47, 48), which could reflect stressors during development. For example, maternal infection and immune activation (MIA) have been implicated in SCZ by epidemiology and animal models (49), but whether MIA causes somatic mutations, and if so in what pattern, are unknown.

The relationship between mutational processes observed here and SCZ might reflect several models. Developmental sSNV may exert direct effects on disease liability analogous to germline mutations: even though only some neurons harbor these variants, the affected population may be large enough to produce phenotypic manifestations. Alternatively, differences in detected somatic variants between SCZ and controls may reflect differences in clonal structures of progenitor populations, producing differential detection sensitivity, akin to focal cortical dysplasia (4). Lastly, various factors involved in SCZ etiology might be mutagenic independent of direct effects on SCZ, manifesting, for example, as enhanced CpG demethylation, inhibition of DNA repair, or decreased removal of mutated cells.

Our data show enrichment of somatic mutagenic processes previously characterized in other contexts, rather than enrichment of functional classes of mutations known to influence SCZ risk in the germline (such as coding mutations). Some somatic variants at TFBS can alter expression of neurodevelopmental genes, favoring models that somatic mutations increase disease liability. Somatic SNVs at TFBS active in development are thus simultaneously products of mutagenesis hotspots and ideal candidates to create risk for developmental brain dysfunction, increasing the probability that variants disrupt transcriptional regulation crucial to neuronal function. They may synergize with germline SCZ risk alleles that typically control gene dosage (1, 16, 17). Finally, the highly recurrent sites impacted suggest that nonspecific mutagenic processes can be channeled by specific TF binding to create recurrent patterns of mutation and potentially increased liability for behaviorally complex phenotypes.

## Materials and Methods

### Sample preparation and sequencing

Frozen post-mortem DLPFC (dorsolateral pre-frontal cortex) pulverized samples of subjects (61 schizophrenic and 25 control) were obtained from the Mount Sinai Brain Bank, part of the NIH NeuroBioBank. All specimens were deidentified, and all research was approved by the Common Mind Consortium. No statistical methods were applied to predetermine sample sizes, but rather we attempted to obtain data from all the affected and control frozen brains available to us at the time of the study and within the budget constraints of the project. Data collection and batching of samples were not randomized. We isolated NeuN+ (Anti-NeuN-Alexa488 (Cat# MAB377X, EMD Millipore) antibody) nuclei from DLPFC tissue samples using fluorescence-activated nuclei sorting (10), followed by standard proteinase-K based DNA isolation with phenol-chloroform cleanup and ethanol precipitation. Sequencing libraries were then prepared with the Illumina TruSeq DNA PCR-free kit, according to the manufacturer’s standard protocol (350bp fragment design). We quantified sequencing libraries using the KAPA Library Quantification Kit (a real-time PCR methodology), and libraries were sequenced at the GeneWiz sequencing facility (NJ, USA) on an Illumina HiSeq X Ten platform, to yield 150bp paired-end reads. Sequencing experiments aimed for a minimum yield of 200x coverage per sample, and the average coverage obtained across all samples was 239x.

### Somatic SNV calling and filtering

Somatic SNVs were identified from WGS sequencing data using the best practices workflow from the Brain Somatic Mosaicism Network (11). Briefly, fastq files were aligned to the GRCh37 reference genome using bwa v0.7.17 (50), and preprocessed using the GATK best practices. Raw variants were then called using GATK Haplotypecaller (51) using a ploidy that corresponds to 20% of the overall sequencing coverage (i.e., ploidy of 50). Variants were then filtered if they fell on genomic regions labeled by 1000 Genomes Strict Mask (11). Variants with a GnomAD (17) population allele frequency >0.001 were filtered as well as variants with variant allele frequencies close to 0.5 (binomial test p < 1e-6) to remove potential germline variants. Candidate sSNVs were required to have >4 independent non-duplicated supporting reads with mapping quality of 20. A panel of normals filter from the 1000 Genomes Project was also used to remove variants that might occur from technical artifacts. The pipeline is readily accessible along with instructions at https://github.com/bsmn/bsmn-pipeline, which was run using the AWS ParallelCluster (https://github.com/aws/aws-parallelcluster) with the following configuration settings (https://github.com/bintriz/bsmn-aws-setup). Variants with VAF > 0.40 were filtered to reduce potential germline variants false positives.

### Somatic copy number variant calling

We performed somatic CNV analysis on 75 samples with mean coverage higher than 100x. We excluded 19 samples (MSSM_033, MSSM_063, MSSM_065, MSSM_069, MSSM_116, MSSM_118, MSSM_158, MSSM_192, MSSM_201, MSSM_266, MSSM_287, MSSM_291, MSSM_293, MSSM_299, MSSM_308, MSSM_309, MSSM_310, MSSM_331, MSSM_338) with coverage less than 100x. In addition, we excluded MSSM_164 with noisy signals in both read depth and allele frequency. Candidates for somatic CNVs were generated by CNVpytor (52) with the caller gathering information from both read depth and split in B-allele frequency of germline SNPs called using GATK haplotype caller run with ploidy=2. Analysis was conducted with two bin sizes: 100 kbps and 10 kbps.

We then applied filters to exclude false positive candidates and germline CNVs. We considered as false positives the following: 1) calls with adjusted p-value from CNVpytor larger than 0.05/(number of samples*3*109/bin size); 2) calls with <50% of well mapped bases (P-bases) as defined by the 1000 Genomes Project; 3) calls with >5% of non-sequenced reference (N-bases); 4) calls only supported by read depth (p-value from BAF signal > 0.01) and with predicted cell frequency <5%; 5) calls with predicted cell frequency <10%; 6) calls found in multiple samples (two calls are considered the same if overlap by 50% reciprocally). We additionally filtered out calls with length <= 3 of bins due to the boundary effects, which may lead to underestimation of cell frequency for germline CNVs.

We were not able to resolve breakpoints for the somatic duplication. Thus, we imputed two haplotypes by phasing germlines SNPs using population haplotypes and then confirmed that the frequencies of the two haplotypes were different.

### Amplicon Validation:

Custom primers were designed for each candidate variant using the default settings in Primer3 (53, 54) to generate 150–300bp amplicons. The primers were commercially synthesized (IDT) and tested on human genomic DNA (Promega) to confirm generation of only one amplicon product at the expected size. Then 10–50ng of genomic DNA from patients (based on sample availability) were used to create amplicons for sequencing, purified using 2X AMPure XP, and run on a gel for quality control. Amplicons from different samples were pooled together and Illumina sequenced to achieve at least 10,000 reads per each unique amplicon. The raw reads were aligned to the reference genome (hg19) and visualized on Integrative Genomics Viewer (IGV) to confirm the presence of each candidate variant. The variant allele frequencies were calculated based on the total number of REF and ALT alleles.

### Variant Annotation

For schizophrenia GWAS loci we used Table S4 of Pardinas (55).

### Schizophrenia Polygenic-Risk-Score calculation

Data from SNP genotype arrays were obtained as previously described (56) and were mapped to the biospecimens in this study with the use of unique CMC individual IDs. Liftover (http://hgdownload.cse.ucsc.edu/admin/exe/) was used to convert marker positions to GRCh38. Markers were then aligned to TOPMed (57) version R2 loci with HRC-1000G-check-bim-v4.3.0 (https://www.well.ox.ac.uk/~wrayner/tools/). HRC-1000G-check-bim-v4.3.0 verifies the marker strand, alleles, position, reference/alternate allele assignments and frequencies and removes A/T & G/C single nucleotide polymorphisms (SNPs) with minor allele frequency (MAF) > 0.4, SNPs with differing alleles, SNPs with > 0.2 allele frequency difference between the genotyped samples and the TOPMed samples, and SNPs not in reference panel. The TOPMed Imputation Server (58) (https://imputation.biodatacatalyst.nhlbi.nih.gov/), which uses Eagle (59) for haplotype phasing, was used for imputation. Variants were filtered and SNPs with imputation R2 > 0.3 were retained. After LD-based pruning of common variants using PLINK2 (60), PLINK2’s implementation of KING (61) was used to estimate relatedness; related samples and samples with cryptic relationships were removed with a kinship coefficient cut-off of ≥ 0.0884. For population stratification, 1000 Genomes (1000G) Project genotypes were lifted-over to GRCh38 and merged with imputed genotypes. Merged genotypes were filtered (retained MAF ≥ 0.01, Hardy-Weinberg equilibrium P value > 1 × 10^−10^, imputation R2 > 0.8), pruned, and principal components were calculated with PLINK2. We used an ellipsoid definition of ancestry (using three standard deviations and three principle components) to select ancestry based on reference superpopulation ancestries in 1000G. PRS-CS (33) was used for polygenic risk score calculation using GWAS summary statistics from the Psychiatric Genomics Consortium (33) with default settings (γ-γ prior=1; parameter b in γ-γ prior=0.5; MCMC iterations=1000; number of burn-in iterations=500; thinning of the Markov chain factor=5). PLINK2 was used to calculate PRS scores on filtered imputed genotypes described above. PRS scores were normalized by scaling to a mean of 0 and a standard deviation (SD) of 1 within the EUR and AFR ancestries to make them easier to interpret.

### Genome-wide somatic mutation burden analysis

For comparisons of the genome-wide sSNV per sample rate in SCZ compared to controls we used a step Negative Binomial regression framework to account for technical and biological covariates as well as overdispersion of the count data. The covariates we controlled for were: ten ancestry principal components, sex assigned at birth, and technical covariates: sequencing center, coverage, year of autopsy, age of death, cause of death, postmortem-interval, or institution where the individual was diagnosed.

Given the large number of covariates for the small sample size, we performed a staged regression approach. To look for the most informative covariates we performed a step negative binomial regression model with the number of sSNV per sample as the outcome variable and all the covariates listed above except for the diagnosis covariate. We used the *step* function in R and the *glm.nb* function from the MASS package. Briefly, the step forward algorithm started with an intercept only model and then computes the Akaike Information criterion (AIC) for each covariate in the regression model and chooses the covariate to add to the model that minimizes the overall model’s AIC at each step until the AIC cannot be improved any more. This approach reduces the risk of overfitting and potential multi-collinearity among the variables. This approach produced coverage and post-mortem interval variables as the most informative.

We then used the covariates from the prior step and added the diagnosis covariate and performed a negative binomial step regression to estimate effect of diagnosis on the sSNV number per sample in cases and controls. Finally, we obtained a null distribution of the diagnosis coefficient by performing bootstrapped step negative binomial regression by permutating the diagnosis labels 10,000 times. After ensuring that the null distribution p-values followed a uniform distribution by qq-plot, we calculated a two-sided bootstrapped p-value by comparing the diagnosis coefficient without permutation to the null, permuted samples.

We developed a power test to estimate the probability to detect significant changes in mutation rates between cases and controls given different magnitudes of mutation rate acceleration in cases versus controls. First, we showed that a negative binomial distribution offers a good fit for the data (see Fig. S1D). Because we have 4-fold more cases than controls we assume that we can estimate parameters of negative binomial distributions for cases and then we will preserve the variance and change the mean for the distribution in controls. Next, we sample 60 cases and 25 controls from corresponding distributions and get negative binomial p-values for the difference between simulated cases and simulated controls. We repeat sampling 500 times for each value of the mean in controls and estimate how frequently out of 500 times the p-value is < 0.05. If the test is unbiased and in line with common sense, for controls sampled from the same distribution as cases, p-values would be <0.05 in 5% of the permutations; indeed, this is the case. Our power test shows that if controls are expected to have two-fold lower mutation rates we will detect differences between cases and controls every time, but if the difference is 1.4-fold we will get significance only 75% of the time (Fig. S1E).

### Mutations in Coding DNA Sequence (CDS)

To calculate number of mutations that fall into CDS we used gencode.v19.annotation.gtf filtering for CDS in protein coding regions.

### Epigenomic mark enrichment

To test the for enrichment of epigenomic tracks (H3K27me3, DHS, H3K4me1, H3K36me3, H3K9me3, H3K4me3) in SCZ cases compared to controls we modeled the number of mutations Y at each track region i as a binomial outcome, such that:

$$Y_{i}\sim Bin\left(S_{i},p_{i}\right)$$

where *S* is the number of sites available to be mutated, and p is the probability of a site being mutated. For each track we constructed a matrix with N, the number of regions, times 2 rows (one for each disease category) and 3 columns (for the intercept, track signal, and diagnosis), so that we can estimate the relationship between each track’s signal and diagnosis status as a log binomial regression:

$$\text{log}\left(p_{i}\right)=\beta_{0}+\beta_{i}\text{log}(\text{score}+1)+\beta_{2}Dx+\beta_{3}\text{log}(\text{score}+1)\cdot Dx$$

where score is the signal of each track respectively, and Dx is the diagnosis status. We considered a result significant if β_3_ ≠ 0, which we interpret as the excess effect of the epigenetic mark on somatic mutation rate in SCZ cases compared to controls. We used the *glm* R package to estimate these parameters. The broadpeak tracks were obtained from Roadmap Epigenomics from sample E081 (18). We also performed the analyses using samples with permuted diagnosis labels to create a negative distribution and observed that the p-value distribution of the diagnosis coefficient followed a uniform distribution with a quantile-quantile plot.

### Transcription factor binding site track

We aggregated the hg19 TFBS bed files from Vorontsov et al (23) using transcription factor tracks with highest reliability and experimental and technical reproducibility (A tracks). Since these tracks are an aggregation across experimental designs, they represent TFBS that are not necessarily tissue-specific.

### DNAse hypersensitivity tracks

We obtained the DHS tracks from ENCODE (62) and Roadmap Epigenome (18). We also obtained tracks from fetal neuron, neuro-progenitor cells, and fetal brain from Girskis et al. (63). For a complete list of the tracks and how to access them see Table S5. For most of the analyses involving DHS we used the broad peak calls with an FDR of 0.01 of fetal brain from sample E081 from Roadmap Epigenome (18) unless otherwise stated.

### Comparison of sSNV rates between cases and controls at active TFBS

We compared the sSNV rates per Mb in a range of +/− 10Kb from the TFBS mid-point. For this analysis the TFBS bed file was filtered by overlaps with the top 10% DHS regions from fetal brain (Table S5) and promoter regions. The promoter regions were defined as 2.5Kb upstream from transcription start sites as defined by *Ensembl* transcripts. The 20Kb range was binned into ~2Kb windows and a Poisson test was used to compare the rates in SCZ and control mutations, using the *genomation* R package (64). We adjusted by the number of samples in each disease category by multiplying the number of sites covered on each bean by the number of cases and controls respectively.

To estimate the significance of the difference in mutation rates for T>G and CpG>GpG mutations at TFBS for SCZ samples and aggregated sets of controls, we first calculated expected numbers of mutations in these regions based on genome-averaged mutation rates in trinucleotide contexts (let us denote these values as $\lambda_{1}$ and $\lambda_{2}$ for cases and controls correspondently). We also know observed numbers of corresponding mutations in these regions n_1_ and n_2_ for cases and controls. Then we ran a binomial test:

$$\text{pbinom}\left(\text{q}={\text{n}}_{2},\;\text{size}={\text{n}}_{1}+{\text{n}}_{2},\;\text{prob}=\lambda_{2}/\left(\lambda_{1}+\lambda_{2}\right),\text{lower}.\text{tail}=\text{TRUE}\right)$$

### Comparison of sSNV rates with genome-wide rates

We compared the sSNV rates per base pair at different distance intervals from the TFBS mid-point. For this analysis the TFBS bed file was filtered by overlaps with the top 5% DHS regions from fetal brain (Table S5) and promoter regions. The promoter regions were defined as 2.5Kb upstream from transcription start sites as defined by *Ensembl* transcripts. The number of mutations from the next interval closest to the TFBS midpoint was subtracted from the subsequent interval to make each interval independent. We used a Poisson test to compare the sSNV rate at each interval, using the genome-wide rate as the expected rate.

### Estimation of mutation rate at mutational hotspots

Our aim is to provide a low bound estimate for the effect of mutational hotspots. The most conservative model to simulate hotspots would be bi-modal mutation rate distribution with one mode corresponding to hypermutable sites and the other mode to remaining sites.

We are relying on two observations:

1. There are overall 266 (without the outlier sample = 250) mosaic T>G mutations in individuals with SCZ
2. There are 3 pairs of T>G mutations that present in two individuals (SVSS)

It is reasonable to assume that mutations are distributed according to a Poisson.

To obtain a conservative estimate for hotspot rate, we assume two different Poisson λ governing mutation rate distribution in the genome:

$\lambda_{1}$ reflects mutation rate in hotspots and $\lambda_{2}$ reflects mutation rate in remaining genome.

The probability to observe double mutants in the dataset:

(1) $$E\left(N_{r}\right)=\frac{\lambda_{1}^{2}*e^{-\lambda_{1}}}{2}*n_{1}+\frac{\lambda_{2}^{2}*e^{-\lambda_{2}}}{2}*n_{2}$$

Nr is the number of recurrent mutations, n_1_ and n_2_ number of hyper mutable and non-hyper mutable sites correspondently. $e^{-\lambda_{1}}$ or $e^{-\lambda_{2}}$ are ~1, because both $\lambda_{1}$ and $\lambda_{2}$ are <<1

In our cohort Nr=3

(2) $$3\approx \frac{\lambda_{1}^{2}}{2}n_{1}+\frac{\lambda_{2}^{2}}{2}n_{2}$$

And because the overall number of mosaic T>G mutations in context of SCZ is 266

$$\lambda_{1}n_{1}+\lambda_{2}n_{2}=266\;n_{1}+n_{2}=1.7*10^{9}\left[\text{number}\;\text{of}\;T/A\;\text{sites}\;\text{in}\;\text{the}\;\text{genome}\right]$$

Now we substitute $\lambda_{1}n_{1}$ with $266-\lambda_{2}n_{2}$, so eq (2) will be:

$$3\approx \left(\lambda_{1}\right)/2266-\left(\lambda_{1}*\lambda_{2}\right)/2{\text{n}}_{2}+\left(\lambda_{2}^{2}\right)/2{\text{n}}_{2},$$

Since $\lambda_{1}>\lambda_{2}$

$$3<\left(\lambda_{1}\right)/2266$$

$$0.027<\lambda_{1}$$

Meanwhile, the genome average is $\bar{\lambda }\sim 1.56*10^{-7}=266/1.7^{*}10^{9}$, thus $\lambda_{1}$ exceeds $\bar{\lambda }$ by a factor of 1.73*10^5^.

### Analysis of cancer data

Cancer sSNVs were downloaded from PCAWG (30). We used tissue-specific DHS tracks from ENCODE (Table S5) to define active TFBS sites in context of specific cancer types.

To calculate recurrence on Fig. 4E, we measured the density of mutations conditioning on the presence of another mutation in position 0 in a different tumor sample. We normalized mutation rate at distance 91–100 nucleotides from focal mutation.

### MPRA library construction

Sequences of 200 base pairs surrounding the somatic variants were obtained from the human hg19 reference genome, with 3 windows designed for each variant: middle – where the variant was placed in the center of the oligo (−99bp/+100bp), left – where the variant was placed towards the 5’ side of the oligo (−59bp/+140bp), and right – where the variant was placed towards the 3’ side of the oligo (−139bp/+60bp). We also included 69 enhancers that were broadly active across 8 cell types from previous experiments as positive controls for the MPRA. Fifteen base pairs of adapter sequences were attached at both ends of the oligos for synthesis: 5′-ACTGGCCGCTTGACG – [oligo sequences] – CACTGCGGCTCCTGC-3′. The oligo library was synthesized by Agilent Technologies.

Following synthesis, 20-base pair barcodes were added to the oligos via a 36 × 50-uL PCR reaction using NEBNext Q5^®^ High-Fidelity 2X Master Mix (M0492L, NEB), with primers MPRA_v3_F and MPRA_v3_20I_R (10 μM concentration for both, see Table S7 for primer sequences) and a 0.5uL template originally resuspended in 100uL for each reaction. The PCR cycle conditions were: 98°C for 2 minutes, 10 cycles (98°C for 10 s, 60°C for 15 s, 72°C for 45 s), and 72°C for 5 min. PCR products were then purified with 1X AMPure SPRIs (Beckman Coulter, A63881) and eluted in 50 μl of water. The MPRA empty vector backbone pGL4:23:DxbaDluc was then digested by SFiI (NEB, R0123S) at 50°C for 1 hour. The cut plasmid backbone and oligo mix were then assembled using the NEBuilder^®^ HiFi DNA Assembly Master Mix (NEB, E2621L) with 2 μg of the cut plasmid and 2.2 μg of oligos, incubated at 50°C for an hour, and subsequently cleaned up with a 1.2X AMPure SPRI, with the final product suspended in 200 μl. 5uL of library assembly was then electroporated into 100 μl of 10-beta Escherichia coli (NEB, C3020K) at 2kV, 200 ohm, 25 mF. The electroporated cells were divided into 10 tubes, each incubated in 1mL of SOC medium at 37°C for an hour before being separately cultured in 20 mL of LB with 100 μg/ml carbenicillin at 37°C for 6.5 hours. At the same time, serial dilutions and spotting plates were conducted to estimate library complexity. The cultures were combined to achieve approximately 1200 colony-forming units per oligo, and the plasmid DNA was extracted using the ZymoPURE^™^ II Plasmid Midiprep Kit.

Then, 20 μg of the resulting vector was then digested with 200 units of AsiSI (R0630L, NEB) in 1x CutSmart buffer in a 600-uL reaction at 37°C overnight. The linearized vector was cleaned up with the Zymo Genomic DNA Clean & Concentrator kit (D4065, Zymo), followed by Gibson assembly with an amplicon containing a minimal promoter, green fluorescent protein (GFP) open reading frame, and partial 3′ untranslated region (3′-UTR). The reaction was conducted with vector:GFP ratio of 1:3.3 at 50°C for 1.5 hours, followed by 1.5X SPRI clean-up. The entire product was then digested again to remove any uncut plasmids with 50 units of AsiSI, 5 units of RecBCD (NEB, M0345S), 10 μg of bovine serum albumin, 1 mM adenosine triphosphate (ATP), and 1X NEB Buffer 4 in a 100-μl reaction at 37°C overnight. The final vector was cleaned with 1.5X SPRI, and electroporated into 10-beta E. coli in 6 batches (2.5uL of plasmid DNA in 50uL cells for each electroporations). Each batch was recovered in 1mL of SOC for 1 hour, then grown in 3 total liter of LB with 100 μg/ml carbenicillin (2mL of recovered cells per liter) for 16 hours at 30°C. The plasmid was pooled and extracted with the Qiagen Gigaprep kit (Qiagen, 12191).

To associate barcodes with oligo sequences, 200ng of the plasmid was amplified using NEBNext Q5^®^ High-Fidelity 2X Master Mix (M0492L, NEB), with primers TruSeq_Universal_Adapter and MPRAv3_a2sa (Table S7) using the following conditions: 95°C for 20s, 5 cycles (95°C for 20s, 62°C for 15s, and 72°C for 30s), and 72°C for 2 minutes. The PCR product was SPRI at 1x and subjected to additional 5 cycles of PCR to attached custom Illumina P5 and P7 indices. Samples were sequenced on a Novaseq S4 flowcell (2 × 150 bp) at the Yale Center for Genome Analysis to achieve a coverage of 10x estimated total number of barcode-oligo sequences. Identification of which barcodes were associated with which oligos was then conducted with the MPRAmatch pipeline (https://github.com/tewhey-lab/MPRAmatch).

### Transfection of MPRA library

Human neuroblastoma SK-N-SH cells (ATCC) were cultured in Eagle’s MEM (EMEM) (ATCC, 30–2003) containing 10% FBS and 1% Pen-Strep. Five total replicates were transfected with the final MPRA library, with each replicate being transfected in different days. 10 million cells of each replicate were trypsinized, resuspended in 400μl of buffer R with 10ug of plasmid library, and electroporated using the Neon Transfection system at 1200 V and 3 20-ms pulses. After transfection, each replicate was recovered in 4 150mm plates 10% FBS-supplemented EMEM without Pen-Strep. After 48hrs, cells were trypsinized, washed with PBS once, flash-frozen using liquid nitrogen and stored at −80°C.

### MPRA RNA sample processing

Total RNA was extracted from the cell pellets with the Qiagen Maxi RNeasy kit (Qiagen, 75162) with on-column DNase digest according to manufacturer’s instructions. A DNase reaction was further performed to remove remaining MPRA library vectors using Turbo DNAse kit (ThermoFisher Scientific, AM2238). The reaction was stopped with 0.1% SDS and 0.05M EDTA. The GFP-transcripts in total RNA were then captured through a hybridization reaction with streptavidin beads (ThermoFisher, 65001) and three GFP-targeted biotinylated oligos (Table S7). RNA was then cleaned up with RNA SPRI (Beckman Coulter, A63987) and converted to cDNA using a Superscript III (ThermoFisher, 18080044) reaction with primer MPRA_v3_Amp2Sc_R (Supplementary Table S7). The relative cDNA abundance was estimated through quantitative PCR along with serial dilutions of plasmid library serving as a standard curve (see Table S7 for primer sequences). The PCR conditions were: 98°C for 30s, 40x of (95°C for 20s, 65°C for 20s, and 72°C for 30s), and 72°C for 2 minutes. To minimize amplification bias, the Ct number reflecting the point at which the amplification just began to take off, subtracted by 1, was used to set up the first PCR for sequencing preparation. cDNA and plasmids were normalized to approximately the same concentration and cycled for 10 cycles using NEBNext Q5^®^ High-Fidelity 2X Master Mix (M0492L, NEB) and primers MPRA_v3_Illumina_GFP_F and TruSeq_Universal_Adapter (Table S7). The product was cleaned up with RNA SPRI at 1X, eluted in 30μL, 20μL of which was then subjected to another round of 6 cycles to attach custom p7 and p5 Illumina adapters with unique sample indices. Samples were sequenced on a NextSeq 2000 platform using the P3 100 cycle kit, with an average of around 100M reads per sample.

### Quantification of somatic variant activity

Oligo counts were obtained via the MPRAcount pipeline (https://github.com/tewhey-lab/MPRA_oligo_barcode_pipeline). Oligos with at least 10 barcodes were retained for analysis and oligo counts were normalized for sequencing depth with the DESeq2 median of ratios method. DESeq2 was then used to estimate the fold change between plasmid DNA and cDNA with Wald’s test and *p*-values were corrected for multiple hypothesis testing by Bonferroni’s method. Significance threshold was determined at adjusted *p*-value less than 0.01 in either the reference or alternate allele in order to call a sequence as having a regulatory effect on expression. For identification of expression-modulating variants, only variants originating from sequences determined to have a regulatory effect were considered. Allelic skew was calculated by comparing the log ratios of the reference and alternative alleles using Wald’s test. All skew p-values were adjusted with the Benjamini-Hochberg procedure and determined to be significant at 5% false discovery rate. The Rscripts for estimating variant activity and allelic skew are available on https://github.com/tewhey-lab/MPRAmodel. Windows of each variant were treated as independent observations. The output from the DESeq2 analysis is reported in Table S8. Difference of odds of emVars stratified by mutational signatures between schizophrenia and cases was calculated using Fisher’s exact test.

### Linking MPRA emVar to SCZ risk genes

SCZ emVars were linked to target genes by predicted gene-enhancer links in human brain tissues and cell types by multiple methods: Activity-By-Contact (ABC) (39, 65), ENCODE-rE2G (66), and Cicero modeling of single-cell ATAC-seq (40). Particularly, ABC gene-enhancer links in human induced pluripotent stem cell (hiPSC) derived bipolar neurons and neural progenitor cells were obtained from https://mitra.stanford.edu/engreitz/oak/public/Nasser2021/AllPredictions.AvgHiC.ABC0.015.minus150.ForABCPaperV3.txt.gz; ENCODE-rE2G gene-enhancer links in adult human brain tissues were obtained from the ENCODE portal https://www.encodeproject.org/; and gene-enhancer links in single-cells human GABAergic and Glutamatergic neurons were obtained from http://catlas.org/catlas_hub/. The list of genes is compiled in Table S9. Summary statistics for all schizophrenia-related GWAS were then downloaded from the GWAS Catalog, filtered to retain SNPs with p-values < 5×10^−8^, and overlapped with SCZ emVars-targeted genes. The potential of SCZ-associated emVars to disrupt or create a transcription factor binding site was evaluated with R package MotifbreakR, with allelic difference p-value cutoff of 1×10^−4^.

## Supplementary Material

Table_S1.csv Table S1: BSMN member names and affiliations.

Table_S2.csv Table S2: Subject clinical and demographic data.

Table_S3.7.18.xlsx Table S3: sSNV mutation call-set, annotated sSNVs, and coding region sSNVs

Table_S4.csv Table S4: Sample covariates for negative binomial regression.

Table_S5.xlsx Table S5: DNAse hypersensitivity track descriptions.

Table_S6.xlsx Table S6: Nearest-gene annotation table of T>G sSNVs.

Table_S7.xlsx Table S7: MPRA primers.

Table_S8.csv Table S8: MPRA results table.

Table_S9.7.18.24.xlsx Table S9: Predicted SCZ emVar targeted genes in human brain tissue.

1

## Data Availability

FASTQ, CRAM, and VCF files were annotated with clinical and sample information and submitted to the NIMH Data Archive into collection C2965 (https://nda.nih.gov/edit_collection.html?id=2965).
