# Supplementary material for: Somatic mosaicism in schizophrenia brains reveals prenatal mutational processes
Source: Science. Author manuscript; Available in PMC 2024 Oct 20. (PMC11490355; doi:10.1126/science.adq1456)
Supplement: 1 [file NIHMS2029380-supplement-1.pdf]

Supplementary Text

Somatic copy number gain in SCZ sample

Somatic CNV (sCNV) calling of our samples revealed a somatic gain in one SCZ case and none in the control samples. We applied CNVpytor (*52*), a method developed to specifically identify sCNVs from high coverage WGS data (see Methods). With this approach we observed one high confidence ~90Kb somatic gain mapping to chromosome 4 in a SCZ sample (Fig. S2A, B). The

somatic gain had an estimated 17.4% mosaic fraction and overlapped intron 1 of the *SORCS2* gene and possibly exon2 (Fig. S2A, B). We attempted to look for split reads supporting the exact breakpoints. Given our ~200x coverage we expected ~16 supporting reads, but were not able to identify them. However, upon closer inspection we found 2 simple repeat regions at both ends of the estimated breakpoints, hindering mappability at the breakpoint loci, thus it is not surprising we were not able to find supportive reads. Nevertheless, the shift in phased-allele frequency provides strong statistical evidence of an event in this region (Fig. S2A). The simple repeat regions suggest that this sCNV potentially arose through tandem duplication. Thus, we expanded the range by 10 Kb around the estimated breakpoints to provide less stringent breakpoints (Fig. S2A).

SORCS2 encodes for a subunit of the sortilin-related VPS10 domain-containing receptor proteins, which are cell-surface proteins implicated in central nervous system development (*67*). Previous GWAS and germline CNV studies have shown that variants in the *SORCS2* locus may confer risk for attention-deficit hyperactive disorder (ADHD), and bipolar disorder (*14, 15*). Similarly, germline SNPs in intron 1 of *SORCS2* have been associated with clinical outcomes in ADHD (*68*), suggesting a potential role in neuropsychiatric disease. Our somatic gain overlaps regions H3K27ac marks present in brain frontal cortex (Fig. S2B), suggesting a potential dysregulation of the expression of this gene by altering enhancer interactions. If the breakpoints actually disrupt exon2, a frame-shift might result in an aberrant protein. However, whether somatic gains within intron 1/exon2 of the *SORCS2* gene plays a role in SCZ requires further functional studies.

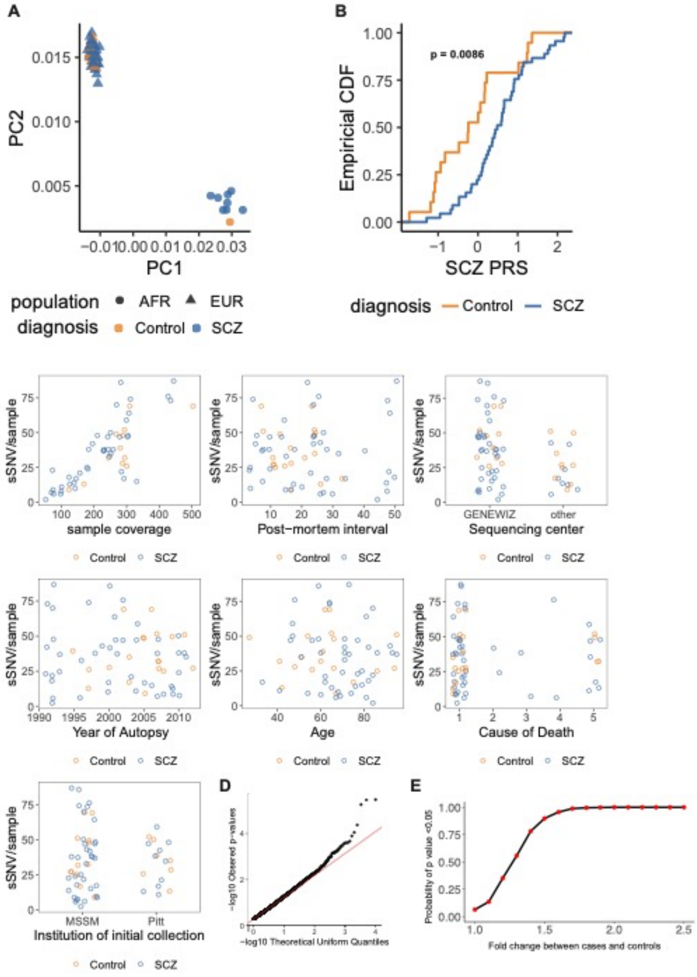

Fig. S1. Population stratification, polygenic risk scores in schizophrenia and control individuals, and technical covariates effect on somatic variants. A) Scatter plot of calculated principal components of genetic ancestry in SCZ cases and controls. Colors indicate diagnosis status and shape indicates population ancestry. B) Empirical CDF curves of normalized (mean= 0; SD =1) polygenic risk scores (PRS) in SCZ cases vs controls colored by diagnosis status. The p-value is calculated by two-sided Kolmogorov-Smirnov test. C) Scatter plots across technical covariates. For categorical covariates a jitter was used across the x-axis for illustration purposes. D) QQ-plot of theoretical uniform distribution and observed null p values of step negative binomial regression for the comparison of genome-wide sSNV in cases compared to controls. E) Power analysis of permutation analysis. At the fold change observed (≤1.2X), permutation test has <25% chance of detecting significant sSNV enrichment in SCZ.

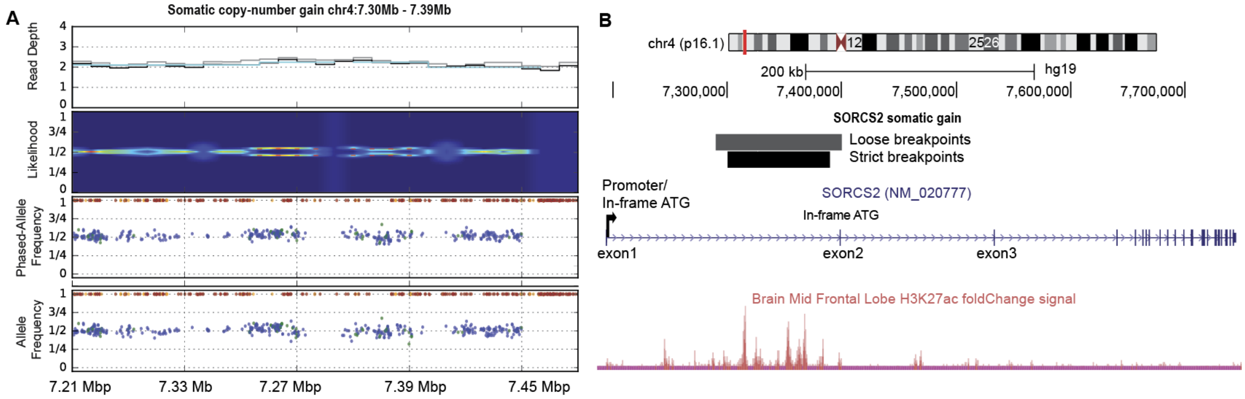

Fig. S2. Somatic copy number gain in a SCZ sample A) Plot of somatic gain. Heatmap represents the likelihood function with the red dotted lines indicated the likely event breakpoints. The dot plots indicate the phased and un-phased SNP allele-frequency respectively. B) A Genome Browser schematic of *SORCS2* gene with H3K27ac histone mark track from Roadmap Epigenomics consortium (*18*).

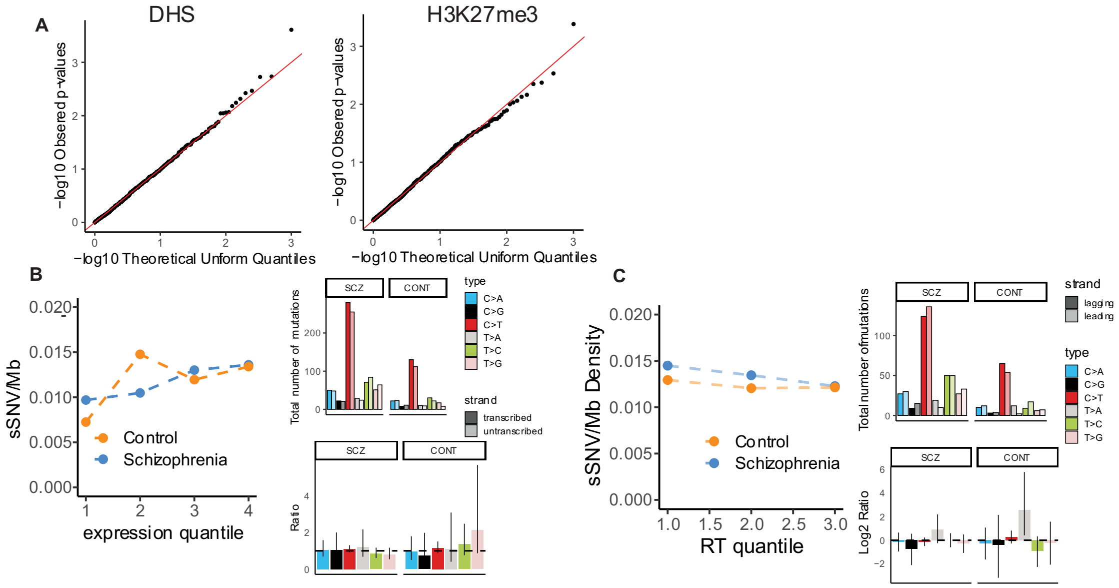

Fig. S3. Calibration of epigenomic tests and characterization of transcriptional and replication strand bias in schizophrenia and controls. A) Examples of qq-plots of empirical null distribution of binomial regression for epigenomic tracks using diagnosis permutation. B) Scatter plot of fetal brain gene expression versus sSNV rate in schizophrenia cases and controls across expression quartiles. Bar plots showing genome-wide transcriptional strand bias as difference in total number of mutations and rate ratio. C) Scatter plot of replication time versus sSNV rate in schizophrenia cases and controls across terciles. Bar plots showing genome-wide replication strand bias as difference in total number of mutations and log2(rate ratio).

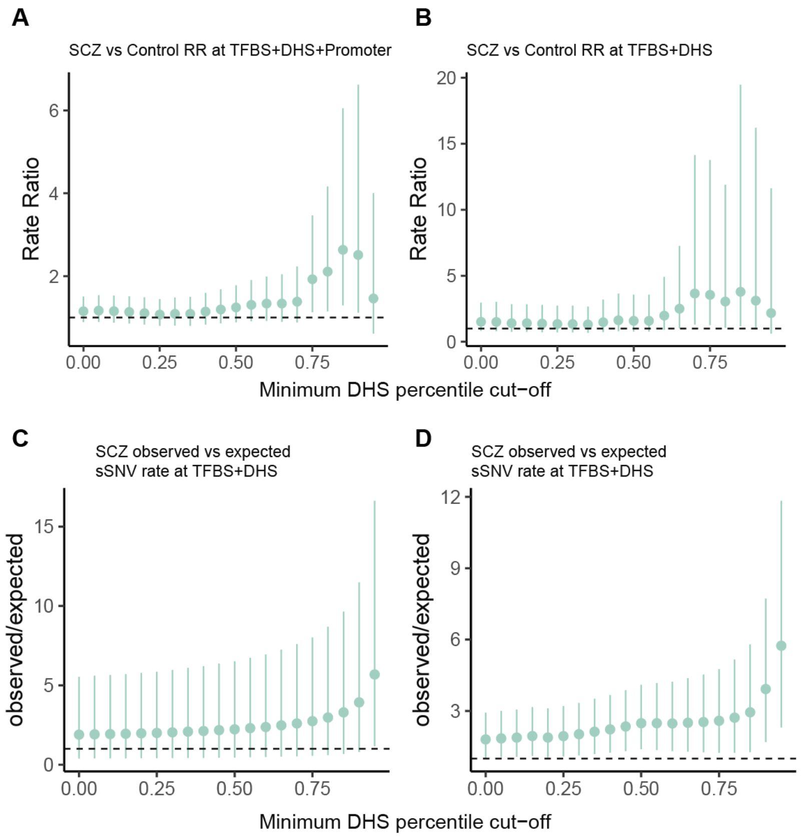

Fig. S4. Enrichment of sSNV at TFBS in SCZ neurons across multiple DHS cut-offs. A, B) Forest plots of rate ratio comparing the mutational rate in TFBS regions close to promoters (A) or not restricted to promoters (B) in SCZ vs controls across multiple DHS percentile cut-offs. C, D) Observed/expected ratio of sSNV at TFBS regions near promoters (C) or not restricted to promoters (D) in SCZ samples across multiple DHS percentile cut-offs. For all plots the lines indicate the 95% Poisson confidence interval.

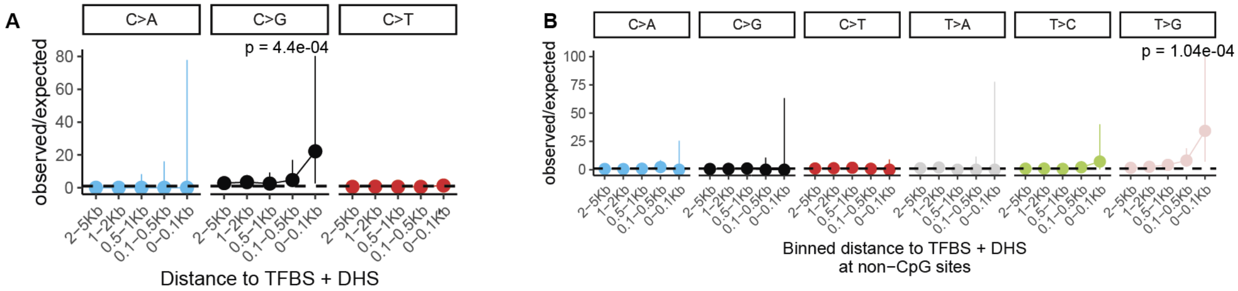

Fig. S5. Relative sSNV rate around active TFBS. A, B) Forest plots of observed vs expected ratios in schizophrenia of different base changes in active TFBS at CpG sites and non-CpG sites, respectively. For panels A, B, p-values and 95% confidence intervals were computed using a Poisson test accounting for trinucleotide context.

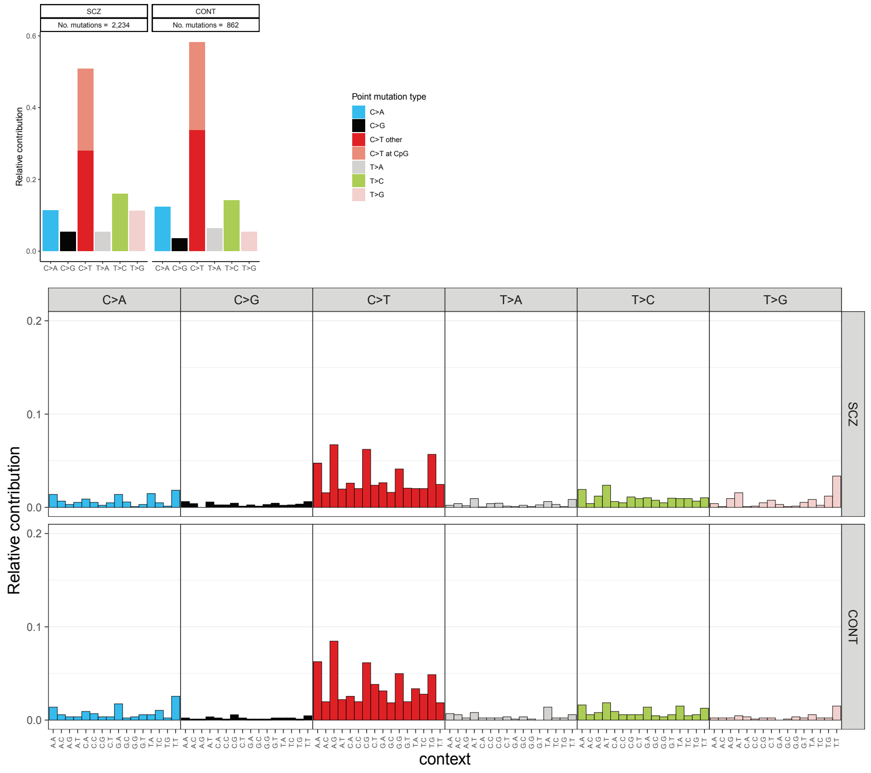

Fig. S6. Single base substitution spectrum in schizophrenia and controls. (Top) Bar plot of single base substitution spectrum in schizophrenia case and controls normalized to the total number of sSNV in each diagnostic category. (Bottom) Bar plots of the trinucleotide context distribution in schizophrenia cases and controls, normalized to the total number of sSNV in each diagnostic category. Legend is for both top and bottom.

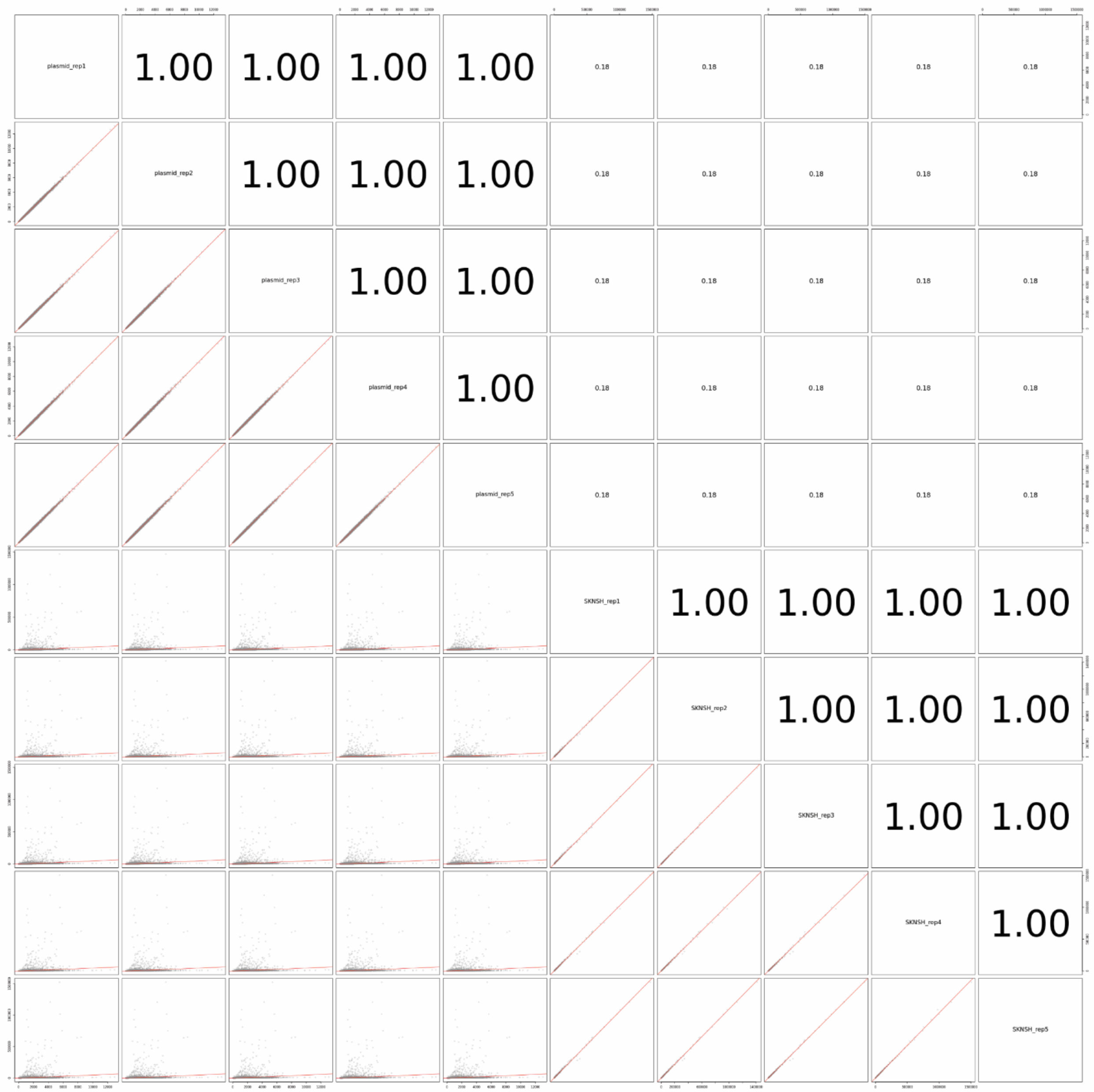

Fig. S7. Reproducibility of MPRA assay. Scatter plot matrix shows high pairwise correlations of barcode sequencing read counts between plasmid (n=5) and RNA replicates (n=5) in the MPRA experiment. Numbers represent Pearson's r.

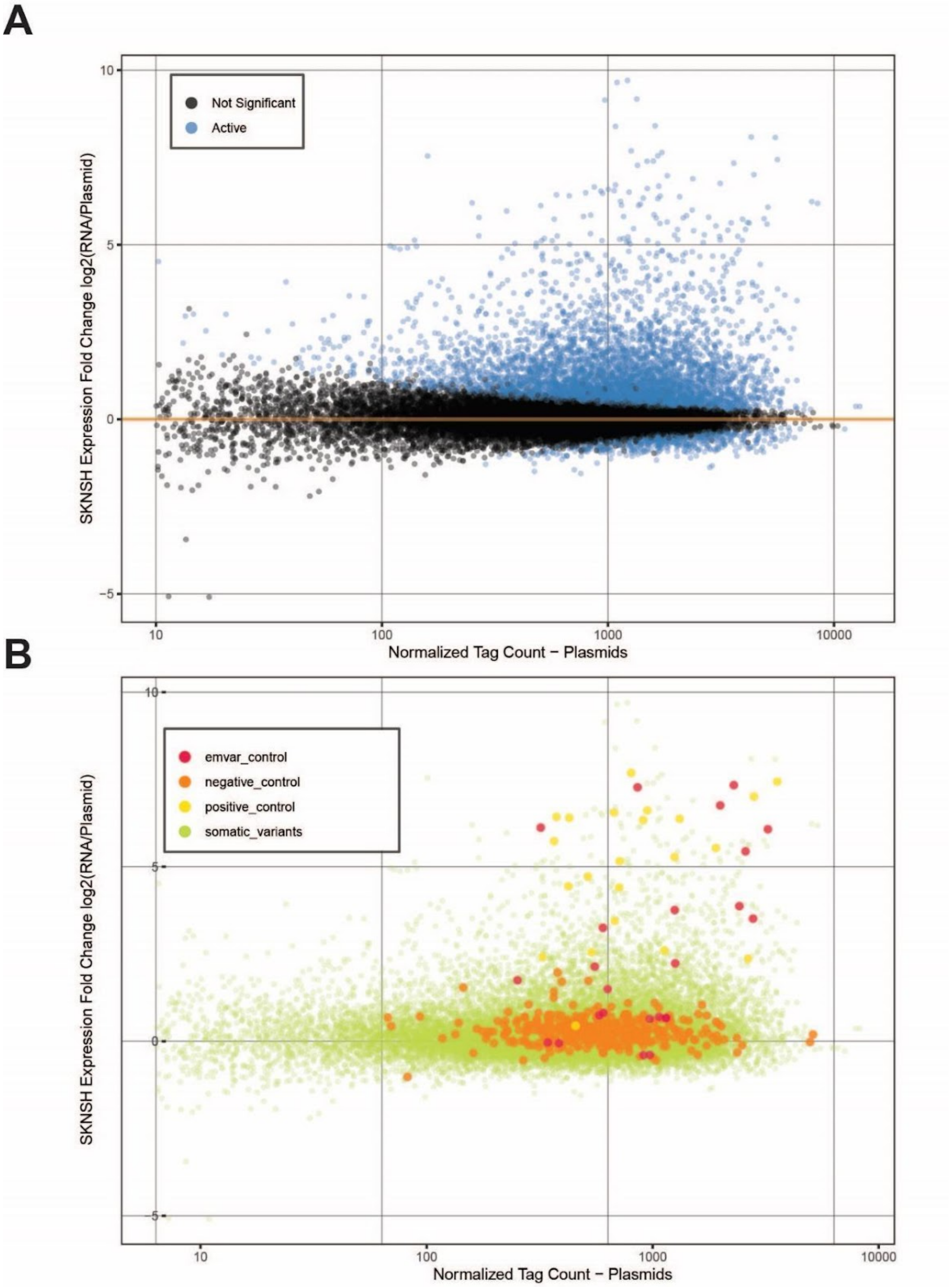

Fig. S8. MPRA controls. (A) Log2 of RNA/plasmid ratio and normalized plasmid barcode counts for active oligos (blue). Significance for active oligo is defined at FDR < 0.01. (B) Log2 RNA/plasmid for variants in the MPRA test set (somatic_variants), negative controls, positive controls, and emVar controls.

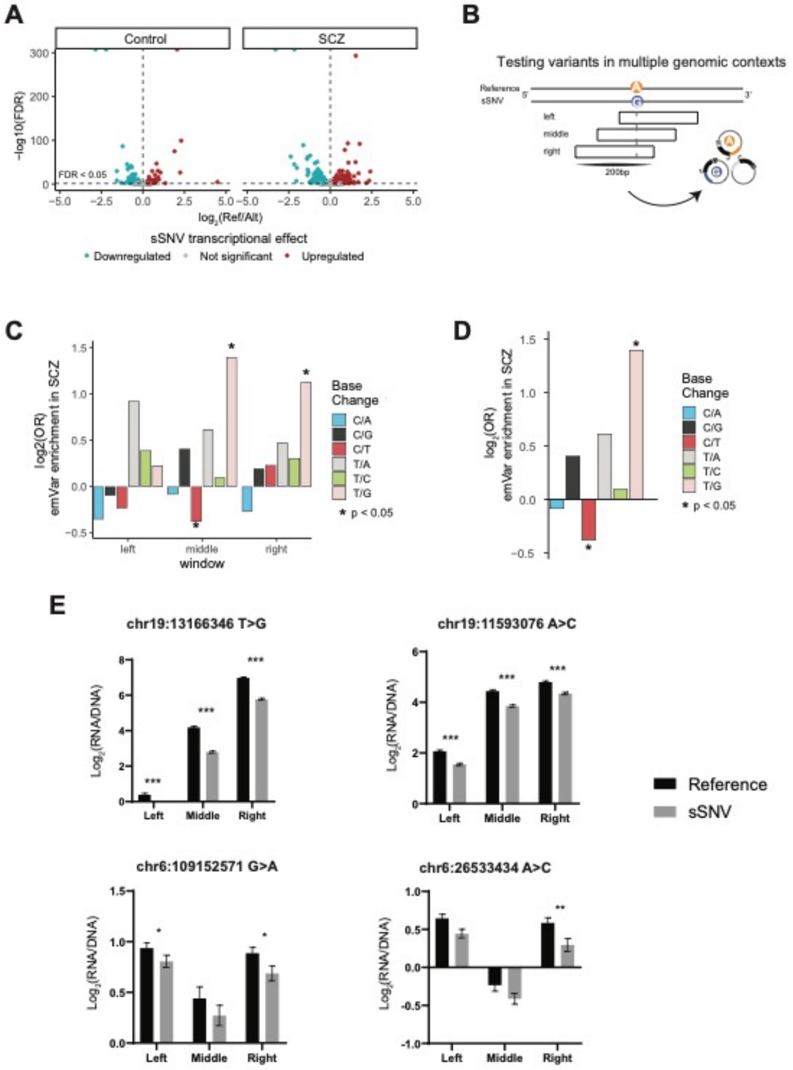

Fig. S9. Overview of MPRA results. A) Volcano plots of effect of sSNV on transcriptional activity in SCZ and control samples. X-axis depicts log2 of activity ratio between reference and somatic allele, with positive values indicating upregulation (red), and negative values indicating downregulation (blue). B) Schematic of MPRA testing of variants within different genomic contexts. C) Bar plots of enrichment of expression modifying variants (emVars) in SCZ vs controls across base changes in different genomic contexts. D) Bar plots of enrichment of emVars in SCZ vs. controls across base changes. P-values were computed using permutation-based Fisher-Exact tests. E) MPRA activity of somatic variants targeting important neurodevelopmental genes and known SCZ risk genes in Fig. 5 demonstrated consistent effect across windows.

Table S1: BSMN member names and affiliations.

Table S2: Subject clinical and demographic data.

Table S3: sSNV mutation call-set, annotated sSNVs, and coding region sSNVs

Table S4: Sample covariates for negative binomial regression.

Table S5: DNAse hypersensitivity track descriptions.

Table S6: Nearest-gene annotation table of T>G sSNVs.

Table S7: MPRA primers.

Table S8: MPRA results table.

Table S9: Predicted SCZ emVar targeted genes in human brain tissue.
